# Colloidal Assemblies Composed of Polymeric Micellar/Emulsified Systems Integrate Cancer Therapy Combining a Tumor-Associated Antigen Vaccine and Chemotherapeutic Regimens

**DOI:** 10.3390/nano11071844

**Published:** 2021-07-16

**Authors:** Chiung-Yi Huang, Shu-Yu Lin, Tsu-An Hsu, Hsing-Pang Hsieh, Ming-Hsi Huang

**Affiliations:** 1 National Institute of Infectious Diseases and Vaccinology, National Health Research Institutes, Miaoli 350, Taiwan; anita9132@nhri.edu.tw; 2 Institute of Biotechnology and Pharmaceutical Research, National Health Research Institutes, Miaoli 350, Taiwan; sylin@bioduro-sundia.com (S.-Y.L.); tsuanhsu@nhri.edu.tw (T.-A.H.); 3 Biomedical Translation Research Center, Academia Sinica, Taipei 115, Taiwan; 4 Graduate Institute of Biomedical Sciences, China Medical University, Taichung 404, Taiwan

**Keywords:** colloidal assemblies, multi-kinase inhibitor, PEG–PLA, tumor-associated antigen

## Abstract

Integrative medicine comprising a tumor-associated antigen vaccine and chemotherapeutic regimens has provided new insights into cancer therapy. In this study, the AB-type diblock copolymers poly(ethylene glycol)–polylactide (PEG–PLA) were subjected to the dispersion of poorly water-soluble molecules in aqueous solutions. The physicochemical behavior of the chemotherapeutic agent DBPR114 in the PEG–PLA-polymeric aqueous solution was investigated by dynamic light scattering (DLS) technology. In vitro cell culture indicated that replacing the organic solvent DMSO with PEG–PLA polymeric micelles could maintain the anti-proliferative effect of DBPR114 on leukemia cell lines. A murine tumor-associated antigen vaccine model was established in tumor-bearing mice to determine the effectiveness of these formulas in inducing tumor regression. The results demonstrated that the therapeutic treatments effectively reinforced each other via co-delivery of antitumor drug/antigen agents to synergistically integrate the efficacy of cancer therapy. Our findings support the potential use of polymeric micellar systems for aqueous solubilization and expansion of antitumor activity intrinsic to DBPR114 and tumor-associated antigen therapy.

## 1. Introduction

Recent achievements in cancer therapy have been driven by integrative medicine, which is composed of chemotherapeutic regimens and tumor-associated antigen therapy that synergistically inhibit oncogenic signaling pathways and train the patients’ immune system to recognize and destroy cancer cells [1,2,3]. However, the path to successful integrative medicine is not straightforward. Chemical incompatibility and immunologic interference are two key challenges that must be overcome when candidate drugs/antigens are blended into one regimen formulation [3]. Clinical investigators must choose from a wide range of options regarding the route, schedule, and sequence of drug/vaccine administration. For feasibility studies of integrative medicine strategies, the first priority is to investigate whether the presence of a delivery system can overcome the chemical incompatibility between drugs/antigens.

Previously, we applied an adjuvantation strategy to engineer amphiphilic bioresorbable polymers as emulsifiers to construct colloidal vesicles in the pursuit of innovative vaccine design [4,5,6]. We proposed that the polymeric nature of the designed emulsifier predominates the stability of the aqueous/oily interfaces during preparation and storage; on the other hand, the degradable nature has a critical influence on the absorbance of the colloidal vesicles post-vaccination [4,5]. However, whether colloidal assemblies comprised of polymeric micellar/emulsified systems can prevent chemical incompatibility between individual drugs/antigens remains unknown.

**Figure 1:**
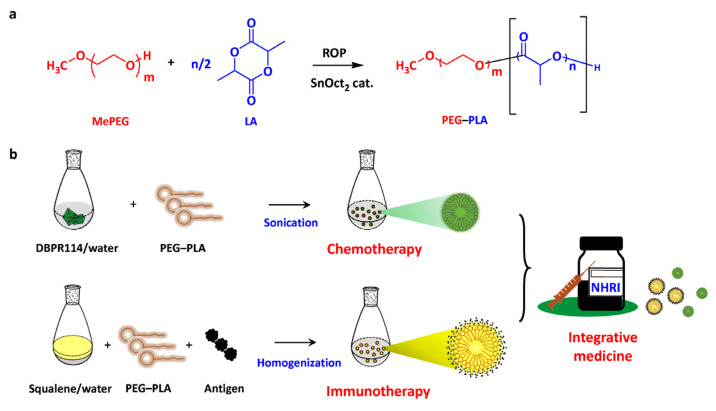
Colloidal assemblies comprised of polymeric micellar/emulsified systems integrate cancer therapy combining a tumor-associated antigen vaccine and chemotherapeutic regimens. (**a**) Synthetic pathway of amphiphilic PEG–PLA copolymer. (**b**) Schematic illustration of PEG–PLA-based colloidal assemblies as a co-delivery system of a tumor-associated antigen and a chemotherapeutic drug. The PEG–PLA polymeric micellar solution was first prepared to serve as a dispersing system for the poorly water-soluble drug, DBPR114. In parallel, polymeric micellar solution mixed with squalene was passed through a homogenizer, rendering a uniform emulsion stock. A mixture of tumor-associated antigens, squalene emulsion and micellar DBPR114 solution can be prepared to treat tumor-bearing mice.

The objective of this study was to combine two therapeutic processes, a tumor-associated antigen vaccine and chemotherapeutic regimens, effectively reinforcing each other via co-delivery of antitumor drug/antigen agents to integrate the efficacy of cancer therapy (Figure 1). First, we conducted a comprehensive analysis to determine the effects of a polymeric micellar delivery system on the disposition of the poorly water-soluble drug DBPR114, a quinazoline-based, multi-kinase inhibitor for the treatment of acute myeloid leukemia (AML) currently formulated in a co-solvent system consisting of DMSO/cremophor EL/saline (10/20/70) prior to intravenous administration [7]. AB-type diblock copolymers made of poly(ethylene glycol)–polylactide (PEG–PLA) were synthesized by ring-opening polymerization of lactide onto the hydroxyl end of methoxy poly(ethylene glycol) (MePEG) [4]. The resulting copolymers were subjected to basic identifications, such as nuclear magnetic resonance (NMR) spectroscopy and gel permeation chromatography (GPC). The physicochemical behavior of DBPR114 in the PEG–PLA-polymeric aqueous solution was investigated by dynamic light scattering (DLS) technology. In vitro cell culture indicated that replacing DMSO with PEG–PLA polymeric micelles could maintain the anti-proliferative activity of DBPR114 in leukemia cell lines. Finally, we plan to conduct a tumor challenge study to gain insight into the whole picture of our drug/vaccine formulations in tumor-bearing mice for the effectiveness of these responses in inducing tumor regression with respect to their therapeutic properties. The results underscore the therapeutic potential of chemotherapeutic drug candidates after formulation with colloidal assemblies, thereby enhancing the efficacy of cancer therapy.

## 2. Materials and Methods

### 2.1. Materials

The AB-type diblock copolymer PEG–PLA was synthesized by ring-opening polymerization of DL-lactide using polyethylene glycol 2000 monomethyl ether (MePEG_2000_) as the macroinitiator and tin(II) 2-ethylhexanoate (SnOct_2_) as the catalyst (Figure 1a), as previously described [4]. The obtained polymers were recovered by re-precipitation using acetone as the solvent and ethanol as the non-solvent, followed by filtration and vacuum drying.

### 2.2. Measurements

^1^H-NMR spectra were recorded at room temperature with a Varian VXR 300 MHz spectrometer (Varian, Palo Alto, CA, USA) using DMSO-*d*_6_ (Sigma, St. Louis, MO, USA) as the solvent. GPC was performed using an outfit composed of an isocratic pump, a refractometer, a PLgel 5-μm mixed-D gel permeation column with length of 300 mm and inner diameter (ID) of 7.5 mm and a PLgel 5-μm guard column with length of 50 mm and ID of 7.5 mm, setting the flow rate at 0.8 mL/min with tetrahydrofuran (THF) as the mobile phase. The number-average molecular weight (*M*_n_) and the polydispersity (*M*_w_/*M*_n_) were expressed relative to the hydrodynamic volume of polystyrene standards (Varian, Inc., Amherst, MA, USA). The particle size of DBPR/polymer in an aqueous solution was monitored by dynamic light scattering (DLS) (NanoBrook 90Plus, Brookhaven, NY, USA).

### 2.3. Cell Viability Assay

The human leukemia cell lines, MOLM-13 and K562, were maintained in RPMI 1640 medium and Iscove’s modified Dulbecco’s medium (IMDM), respectively. Both media were supplemented with 10% fetal bovine serum (FBS), 10 U/mL penicillin, and 10 g/mL streptomycin. Cell suspensions (10^4^ cells per mL) were cultured in a 96-well culture plate at 37 °C and in 5% CO_2_ for 16 h; subsequently, the cells were treated with DBPR114 in the presence or absence of a polymeric vehicle. After 72 h, cell viability was assayed with a Cell Counting Kit-8 (CCK-8, Dojindo, Rockville, MD, USA) according to the supplier’s instructions. The half-maximal effective concentration (EC_50_) value was defined as the concentration of compound (DBPR114 or polymeric vesicles) that caused a 50% reduction between the baseline and maximum in cell viability and was determined with serial two-fold dilution concentrations ranging from 38 to 0.296 nM for MOLM-13 and from 38,000 to 1187.5 nM for K562.

### 2.4. Ethic Statement

All experiments were conducted in accordance with the guidelines of the Laboratory Animal Center of NHRI, and the protocol was approved by the NHRI Institutional Animal Care and Use Committee (NHRI-IACUC-107149-M1-A-S01).

### 2.5. Integrative Medicine

We plan to construct an in situ carcinoma tumor-associated antigen therapy model consisting of ovalbumin (OVA) protein as a tumor antigen and EG7 (a thymoma cell line, EL4, transfected with OVA complementary DNA) cells as tumor cells [6]. A total of 2 × 10^5^ EG7 tumor cells per mouse were first inoculated s.c. into the flanks of C57BL/6 mice (*n* = 5 per group). Upon the appearance of palpable tumors, the mice were subcutaneously injected with one shot near the tumor inoculation site with 100 µL of squalene emulsion containing 600 µg of PEG–PLA polymer (sample P1) and 7 µL of squalene oil suspended in pure water [4], either blank or formulated with 10 µg of OVA. Another treatment group was administered 100 µg of DBPR114-polymeric micelles (DBPR114/P1 of 1/100 w/w) in an OVA protein-formulated emulsion. Tumor sizes were measured twice per week using digimatic calipers. Tumor volumes were calculated according to the following formula: (length × width × width)/2.

## 3. Results

### 3.1. Characterization of PEG–PLA by NMR, GPC and Aqueous Solubilization

An AB-type diblock copolymer (sample P1) consisting of hydrophilic block PEG with a molecular weight of 2000 Da and an equivalent molecular weight of lipophilic block PLA was designed and synthesized by ring-opening polymerization. By tailoring the PLA chain length (sample P2), the hydrophilic-lipophilic ability could easily be manipulated. Generally, the hydrophilic-lipophilic balance of the copolymer strongly influences the relative affinity between water and water-immiscible molecules, leading to optimization of the micellar system. Table 1 shows the molecular characteristics of the PEG–PLA copolymers considered in this study. The PEG/PLA weight ratios were calculated from the integration of ^1^H NMR signals belonging to PEG blocks at 3.6 ppm and to PLA blocks at 1.5 ppm, as described previously [4]. The molecular weight distribution of sample P2 was unimodal with a low polydispersity index (*M*_w_/*M*_n_ = 1.12); however, sample P1 exhibited a rather broad distribution (*M*_w_/*M*_n_ = 1.23).

Amphiphilic block copolymers generally self-assemble into core-shell micellar architecture in aqueous solution, with a core consisting of hydrophobic blocks (PLA) and a shell composed of hydrophilic chains (PEG) after vortexing and sonication. DLS measurements were performed to confirm aqueous solubilization. The data showed that the PEG–PLA (sample P1) polymeric aqueous solution contained micelles with an average diameter of 60 ± 10 nm. Tailoring the PLA chain length (sample P2) slightly increased the size of the polymeric micelles (185 ± 60 nm). Conversely, DBPR114 was not well-dissolved but was suspended in water at room temperature (1500 ± 300 nm). It appears that co-incubation of DBPR114 with PEG–PLA polymer (sample P1) in aqueous solution possessed a unimodal distribution, with an average diameter of approximately 50 nm, indicating that micellization of DBPR114 to polymeric micellar carriers provides an approach to aqueous solubilization.

### 3.2. Anti-Proliferative Activity of DBPR114 in Leukemia Cell Lines

In a previous study, DBPR114 was proven to be a potent multi-kinase inhibitor for the treatment of AML [7]. In this study, the anti-proliferative activity of DBPR114 was determined to inhibit proliferation against MOLM-13, an FLT3-ITD mutation-positive AML cell line [7]. As shown in Table 2, a formulation of DBPR114/DMSO could induce high-level anti-proliferative activity in MOLM-13; however, replacing the DMSO solvent with water attenuated DBPR114 activity in vitro. In contrast, replacing DMSO with PEG–PLA polymeric micelles could maintain DBPR114 activity. Our findings support the potential use of polymeric micellar systems for aqueous solubilization and the anti-proliferative activity of DBPR114. However, the anti-proliferative activity was in the low micromolar range in K562 cells, an FLT3-negative leukemia cell line, suggesting that DBPR114 could act via the FLT3 pathway. It should be noted that PEG–PLA polymeric micelles failed to inhibit the proliferation of MOLM-13 and K562 cells in vitro, indicating that the polymers were biologically inert in the cell lines tested.

### 3.3. Co-Delivery of Antitumor Drug/Antigen Agents Enhances Antitumor Efficacy

We plan to evaluate the efficacy of colloidal assemblies as a co-delivery system of a tumor-associated antigen and a chemotherapeutic drug (Figure 1b). As mentioned above, a polymeric micellar solution containing PEG–PLA (sample P1) in water was first prepared to serve as a dispersing system for the poorly water-soluble chemotherapeutic drug, DBPR114. Conversely, a polymeric micellar solution mixed with squalene was passed through a homogenizer, rendering a uniform emulsion stock. A mixture of tumor-associated antigen, squalene emulsion and micellar DBPR114 solution was prepared to treat the tumor-bearing mice.

**Figure 2:**
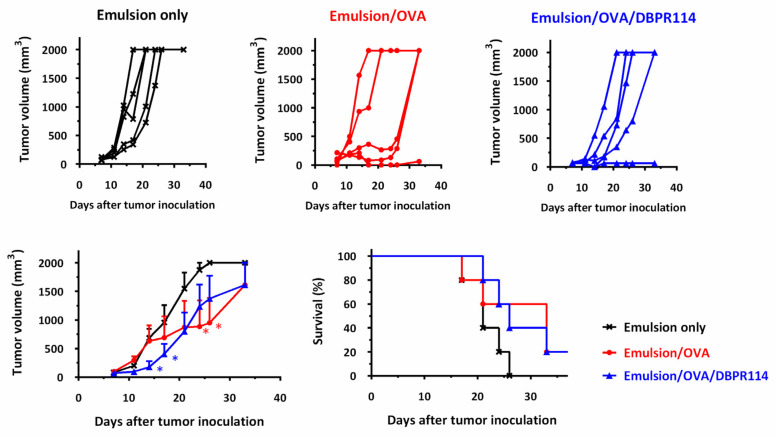
Antitumor efficacy of co-delivery of OVA/DBPR114 to C57BL/6 mice bearing EG7 tumor cells. Mice were inoculated s.c. in the flank with EG7 tumor cells (2 × 10^5^ cells/mouse). Upon the appearance of palpable tumors, five mice per group were treated with emulsion alone or emulsion formulated with OVA protein in the absence or presence of DBPR114 micelles. Tumor volumes in tumor-bearing mice were monitored twice per week and recorded individually. The mice were euthanized when the tumor volume was larger than 2000 mm^3^. The data are expressed as the mean value with standard error of the mean. The tumor volumes were compared by performing a Student’s *t*-test. * *p* < 0.05 compared with the group of emulsions only.

We applied a cancer immunotherapy model consisting of OVA protein and EG7 cells as the tumor-associated antigen and targeted tumor cells, respectively [6]. C57BL/6 mice were first inoculated with EG7 tumor cells. At day 7, the tumor-bearing mice were treated with three strategies, including carrier alone (squalene emulsion), tumor-associated antigen therapy (emulsion-formulated OVA protein), and tumor-associated antigen plus chemotherapeutic agent (emulsion-formulated OVA protein with DBPR114). The tumor volume in each individual mouse and the survival rate of the mice were recorded, as shown in Figure 2. No protection was observed in the mice that received the emulsion-treated control; in this case, the tumors grew progressively. The mice started to die at day 17, and all of the mice died before day 21. A single injection with emulsion plus tumor-associated antigen (OVA) provided a better protective effect than emulsion. Interestingly, additional supplementation of DBPR114 micelles was able to further slow tumor growth within 21 days; beyond, the incorporation of DBPR114 cannot give enough tumor regression in thymoma EG7 cell line. It should be noted that a single treatment of DBPR114 is a multi-kinase inhibitor targeted for AML cell line through the FLT3 pathway, and the low-level anti-proliferative activity was detected in an FLT3-negative leukemia cell line [7]. Here, we demonstrated that DBPR114 could play an auxiliary role in the expansion of antitumor activity intrinsic to tumor-associated antigen therapy. However, the inoculated EG7 cells were not eliminated in the mice that received a single injection of the designed formulations; the dosage and frequency must still be optimized.

## 4. Discussion

Therapeutic vaccines or immunotherapy refers to the clinical manipulation of the immune system with the goal of treating a disease [8]. For example, cancer vaccines that contain tumor-associated antigens are applied to harness the body’s natural immune responses to fight malignant cells, thus bypassing immunosuppression and immune evasion mediated by cancer growth and metastasis [8,9]. To advance immune-based therapeutic strategies, it is important to understand that the generation of an effective antitumor immune response necessitates the sequence of events referred to as the tumor-immunity cycle [10,11]. Protective tumor immunity is generally believed to require the stimulation of innate immune responses, which provide tumor antigen presentation, mature APCs and activated NK cells; induction of potent cancer-specific CTL activities and antibodies; and modulation of the immunosuppressive tumor microenvironment.

Cytotoxic chemotherapy and targeted therapies are two major pillars of chemotherapeutic agents [7,12]. Cytotoxic chemotherapy works mainly by interfering with cell division (mitosis) and can be classified as: (1) alkylating agents, which stop tumor growth by crosslinking guanine nucleobases in DNA double-helix strands; (2) antimicrotubule agents, which block cell division by preventing microtubule function; (3) topoisomerase inhibitors, which block growth signals associated with receptor tyrosine kinases; (4) cytotoxic antibiotics, which interrupt cell division; and (5) antimetabolites, which impede DNA and RNA synthesis. On the other hand, targeted therapies aim at receptors, ligands, or intracellular molecules involved in the signal transduction of cancer cells. A chemotherapeutic regimen for DBPR114 in a mouse model was proposed to be injected intravenously once daily for two continuous weeks [7].

This study focuses on the investigation of polymeric colloidal assemblies associated with aqueous solubilization of a poor water-soluble agent and reinforcement of antitumor activity intrinsic to tumor-associated antigen therapy. We proposed/demonstrated that polymeric colloidal assemblies comprised of micellar/emulsified systems could overcome chemical incompatibility between a model tumor-associated antigen and a chemotherapeutic agent. A single injection composed of a tumor-associated antigen vaccine and chemotherapeutic agent slowed tumor growth compared to an emulsion-treated control and a tumor-associated antigen alone. However, immunologic interference between antigens/drugs also plays a critical role in integrative medicine. It appears that there is a lack of synergistic efficacy when tumor-associated antigen vaccines are combined with certain chemotherapies due to potent immunosuppressive effects, usually associated with patients treated with these chemotherapeutic agents [12,13]. It has also been reported that tumor-associated fibroblasts play a critical role in the suppressive immune tumor microenvironment [14]. To unleash antitumor immunity, further research must be conducted to overcome the limitations in achieving a reverse immunosuppressive tumor microenvironment and to facilitate therapeutic vaccination in desmoplastic tumors. With the aim of extending our results to enhance the efficacy of immunotherapy candidates in clinical applications, the research setting is rationally designed to investigate the impact of antitumor drug/antigen agents and residue compounds issued from the degradation of polymeric vesicles on the enrichment of the immune microenvironments at local injection tissues, ipsilateral draining lymph nodes and tumor bed. We will also investigate whether the co-delivery of drug/vaccine candidates with optimal formulations can rephrase tumor cell susceptibility to cytotoxic T lymphocytes (CTL)-mediated killing towards the enrichment of the tumor immune microenvironment for tumor-bearing mice. These experiments require further assessment of parameters tuning in a small pilot scale high-shear fluid process to elaborate sufficient amounts of samples with consistent and reproducible properties.

## 5. Conclusions

In the present study, we proposed/demonstrated that polymeric colloidal assemblies comprised of micellar/emulsified systems could overcome chemical incompatibility between a model tumor-associated antigen and a chemotherapeutic agent. Our findings support the potential use of polymeric micellar systems for aqueous solubilization and expansion of antitumor activity intrinsic to tumor-associated antigen therapy. Adjuvant discovery using platform technology is very important for enhancing the potency of integrative medicine comprised of a tumor-associated antigen vaccine and chemotherapeutic candidates.

## Figures and Tables

**Table 1:** Compositional and molecular characteristics of the designed PEG–PLA copolymers.

Sample	^1^H NMR ^a)^		GPC ^b)^		Particle Analysis in Water ^c)^			
					Size (nm)		Number (kcps)	
	W_PEG_/W_PLA_	*M*_n_	*M*_n_	*M*_w_/*M*_n_	Blank	With DBPR114	Blank	With DBPR114
P1	50:50	4000	4500	1.23	60 ± 10	50 ± 10	1400 ± 85	1050 ± 240
P2	70:30	2800	3100	1.12	185 ± 60	5950 ± 1100	450 ± 30	320 ± 40

^a)^ Calculated by ^1^H NMR; ^b)^ determined by GPC in THF; ^c)^ monitored by DLS (mean ± STD).

**Table 2:** The anti-proliferative activity of DBPR114 on leukemia cell lines.

	Cell Line	EC_50_ (nM) ^a)^	
Formulations		MOLM-13 (Heterozygous)	K562 (Control Group)
P1		>50,000	>50,000
P2		>80,000	>80,000
DBPR114/DMSO		1.05	7620
DBPR114/water		10	9821
DBPR114/P1		1.6	4615
DBPR114/P2		1.5	7110

^a)^ The EC_50_ value was read as the concentration of the candidate compound that caused a 50% reduction between the baseline and maximum cell viability.
